# Management of medically unexplained symptoms: outcomes of a specialist liaison clinic

**DOI:** 10.1192/pb.bp.112.040733

**Published:** 2014-06

**Authors:** Frank Röhricht, Thomas Elanjithara

**Affiliations:** 1 East London NHS Foundation Trust and University of Essex; 2 Institute of Psychiatry, London

## Abstract

**Aims and method** Service utilisation and clinical outcomes of a newly developed specialist primary-secondary care liaison clinic for patients with medically unexplained symptoms (MUS) were evaluated in a cross-sectional and feasibility pilot study. The impact of body-oriented psychological therapy (BOPT) was explored in a small cohort of patients with an identified somatoform disorder.

**Results** Of 147 consecutive referrals, 113 patients engaged with the assessment process. Of patients with MUS, 42% (*n* = 45) had a primary diagnosis of somatoform disorder, 36% (*n* = 38) depressive disorder, and depressive symptoms (even subsyndromal) mediated the effect of somatic symptoms. A marked variation of presenting complaints and service utilisation across ethnic groups was noted. A significant reduction in somatic symptom levels and service utilisation was achieved for patients undergoing BOPT.

**Clinical implications** A high proportion of patients with MUS have undiagnosed and therefore untreated mental disorders. New and locally derived collaborative care models of active engagement in primary care settings are required. Patients with somatoform disorder may benefit from BOPT; this requires further evaluation in adequately powered clinical trials.

Medically unexplained symptoms (MUS) is the term used to address disorders where physical symptoms have no medical explanation.^1^ Currently, most patients with general MUS fit the diagnostic criteria for ‘undifferentiated somatoform disorder’ (DSM-IV, 300.82; ICD-10, F45.1).^2,3^

According to data on healthcare utilisation, 15-30% of consultations in primary and secondary care concern patients with MUS (e.g. chronic pain, fatigue, headache, dizziness or functional somatoform disorder),^4-6^ resulting in significantly higher healthcare utilisation in secondary care^7^ and high associated costs.^8,9^ A number of models for engaging patients with MUS in primary care have been used,^10^ but those are limited by poor acceptability among general practitioners (GPs) and patients alike.^11^ Specialist secondary mental healthcare services with their focus on severe mental illness rarely make provisions for these patients. Systematic studies suggest that these patients have high psychiatric comorbidity, severe functional impairment and a poor quality of life; typically patients with MUS see themselves as physically ill.^11-13^

Smith & Dwamena^5^ emphasised the lack of evidence-based treatment guidelines for primary care clinicians who care for most patients with MUS. An increase in the number and breadth of therapies for this patient group is therefore desirable. Previous research has demonstrated that a significant improvement in therapeutic engagement and symptom reduction can be achieved while offering the symptom-focused approach of a body-oriented psychological therapy (BOPT) for specific psychosomatic disorders.^14-16^

This evaluation aims to:

1. analyse the clinical characteristics and service utilisation of patients referred with MUS;
2. study the impact of somatic and depressive symptoms on patient function;
3. explore service use and distress levels of patients with somatoform disorder both before and after BOPT.

## Method

A cross-sectional analysis was conducted on data obtained from patients referred consecutively to a specialist MUS clinic in the London borough of Newham (70% of the population are derived from minority ethnic groups; sixth most deprived borough in the country).^17^ The clinic was developed following a consultation process involving the GP mental health leads and used existing secondary care resources only. It aimed to provide an assessment and consultation service for patients with a variety of different MUS (referral criteria in Box 1), who would otherwise have no access to a local primary or secondary healthcare service specific to their needs. Clinical interviews/assessments were carried out by two trained psychiatrists over a period of 24 months; primary/secondary diagnoses and symptom severity were established while applying ICD-10 criteria and using a range of structured assessment tools.

**Box 1** Referral criteria for the MUS clinic

Patients aged 18-65 presenting persistently with physical symptoms, often persistent requests for medical investigations, in spite of repeated negative findings (where no physical basis for the symptoms was established/no evidence for disturbance of structure or function of the physical system/organ concerned). The clinic provides specialist assessments for the following group of patients (all of whom must have a score of ⩾10 on the Patient Health Questionnaire (PHQ-15) somatic symptom severity screening scale or one very prominent medically unexplained symptom (e.g. pain):

- patients who frequently present in primary or secondary care with medically unexplained somatic sensations
- patients with a suspected or established diagnosis of severe and enduring conversion or somatoform disorder
- patients with an established or suspected diagnosis of body image disorder
- patients with a suspected or established diagnosis of anxiety and/or depressive disorder, who mainly present with predominant somatic symptoms.

Patients with an established diagnosis of somatoform disorder were offered body-oriented group psychotherapy. The BOPT approach combines verbal with non-verbal strategies with a focus towards emotional processing/expressiveness, movement behaviour and body/self-perception; it is regarded as advantageous for patients with MUS since the bodily complaints remain the focus of therapy. The therapy is not aiming to ‘eradicate’ symptoms but to work with and through the body in respect of mental distress associated with the symptoms. The interventions do not directly address psychological processes associated with bodily experiences, aiming at a more subtle integration of the somatic and psychological aspects.

The baseline analysis included all 147 referrals from the first 2 years of the clinic (mean age 41.3 years, s.d. = 10.9; 32.6% male, 67.4% female). Patients were grouped for analysis according to their ethnicity (African-Caribbean: 11.5%, *n* = 15; Asian - including Indian, Pakistani and Bangladeshi: 38.9%, *n* = 50; and White: 31.1%, *n* = 39; Other: 18.5%, *n* = 23; and remaining - missing data) and current primary diagnosis according to ICD-10 criteria: somatoform disorder (F45), dissociative disorder (F44), depressive disorder (currently in depressive episode; F32-34), anxiety disorder (F40-43) and other disorders (all other diagnoses). Comorbid diagnoses were also recorded.

Service utilisation at baseline was recorded as: (a) number of attendances at the GP practice; (b) number of emergency service presentations (A&E); and (c) referrals made to other specialties in the 12 months prior to the MUS clinic referral. Clinical and functional morbidity were assessed with the: Hamilton Rating Scale for Depression (HRSD; with cut-off points of 15-19 for moderate, 20 or above for severe);^18^ Patient Health Questionnaire (PHQ-15);^19^ Global Assessment of Functioning (GAF);^20^ and Screening for Somatoform Symptoms-7 (SOMS-7).^21^

Following the descriptive analysis of service utilisation and morbidity scores, further analysis was carried out to identify differences between diagnostic and ethnic groups in respect of somatic symptoms and other clinical characteristics. This was conducted with univariate analysis using student’s *t*-test or ANOVA for quantitative variables or χ^2^-test for categorical variables. Predictive factors were assessed with variables entered into multiple logistic regressions with symptom scores or service utilisation as the dependant variable. Changes in symptom score and data on service utilisation for 3 months after the treatment with BOPT ended were analysed using paired *t*-tests. Statistical analysis used SPSS 17 for Windows. Analyses were only carried out if the missing data were less than 30% of the numbers.

## Results

### Referrals

A total of 147 patients were referred to the clinic. Most referrals came from GPs (*n* = 65, 44.2%) and fellow mental health professionals (*n* = 59, 40.1%). The remaining 23 patients were referred by specialist medical services from the general hospital. There was a notable difference in referral rates across GP surgeries in the borough, with only half of practices making any referrals (mostly 1-3 patients per GP practice in total).

### Somatic symptoms/complaints analysis

**Fig 1:**
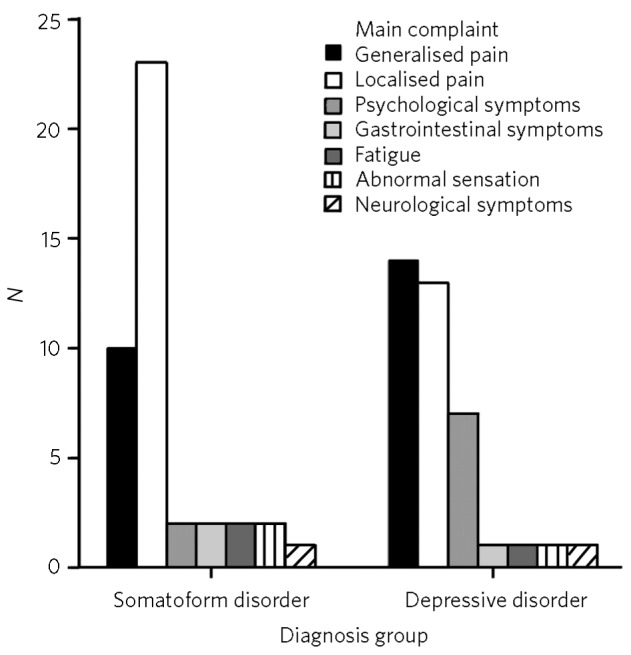
Main presenting complaint for somatoform and depressive disorder diagnosis groups.

The most frequent presenting complaint was localised pain (e.g. headache, pain in legs or back; *n* = 44, 38.9%), followed by generalised pain (*n* = 31, 27.4%); 7 patients (6.2%) reported neurological symptoms (e.g. paralysis, loss of sensation). Only 13 patients (11.5%) reported psychological symptoms (such as feeling low, anxious) as their main presenting complaint (Fig. 1 presents most frequent complaints for two disorder groups, depressive and somatoform disorders).

Among patients with a current depressive episode, only 7 (18.4%) reported psychological symptoms (low mood) as their presenting complaint. Almost all patients presented with multiple physical complaints; 32% of females (*n* = 25) and 15% of males (*n* = 6) reported generalised pain. Asian patients reported generalised pain more frequently than all the other ethnic groups together as their main presenting complaint (37%). A significantly higher proportion of patients in the White group (23%) reported psychological symptoms as their presenting complaint (compared with only 3% for the rest of the cohort, Pearson’s χ^2^ = 7, d.f. = 1, *P* = 0.03).

### Analysis of diagnostic groups

The majority of patients assessed at the MUS clinic (*n* = 106 out of 113) received a diagnosis of mental disorder (Table 1); there was no significant between-group difference according to ethnicity. Nearly a third of patients (*n* = 30, 29%) fulfilled criteria for a comorbid diagnosis, predominantly depressive disorder (*n* = 20) or anxiety disorder (*n* = 10). The majority of patients diagnosed with a current depressive episode (*n* = 26, 68.4%) did not have a recorded history of depression.

### Impact of somatic and depressive symptoms on patient functioning

In the somatoform disorder group, HRSD and SOMS-7 scores were negatively correlated with GAF scores (Pearson’s correlation *r* = –0.68, *P*<0.001 and *r* = –0.62, *P*<0.001 respectively). There was a positive correlation between HRSD and SOMS-7 total scores in this group (*r* = –0.65, *P*<0.01). Entering HRSD and SOMS-7 scores along with age, gender, ethnicity, comorbidity and medication use into a multiple regression model, HRSD score was the only significant parameter associated with the level of function (*F* = 11.2, *t* = –2.5, *P*<0.05). Similar associations were not observed in other diagnostic groups. In the depressive disorder group, only the HRSD scores were negatively correlated with the GAF score (*r* = –0.4, *P*<0.05), but no correlation was found between the HRSD and SOMS-7 scores.

### Service utilisation prior to referral to MUS clinic

#### GP surgery attendance

The mean number of attendances at a GP surgery in the previous year for the MUS cohort was 13.6 (s.d. = 10.5, range 0-45). Female patients (mean = 15.8 *v*. male patients: mean 9.6; *F* = 6.2, d.f. = 1, *P*<0.05) and Asian patients (mean = 16.5 *v*. others: mean = 11.5; *F* = 4.38, d.f. = 1, *P*<0.05) attended their GP practice significantly more often.

#### A&E attendance and referrals to other specialties

Mean number of attendances at the A&E department of the local general hospital during the 12 months prior to referral was 2.5 (s.d. = 3.5, range 0-16). There was no significant association between the number of A&E attendances and any other factors. The mean number of referrals to specialist services was 2.7 (s.d. = 2.4, range 0-12). Significantly more referrals were recorded for: females, individuals with medical history of physical disorder and those of White ethnicity. A significantly lower number of referrals to specialist services was recorded for Asian patients when compared with the sample as a whole.

### Utilisation of MUS clinic service

Overall, 34 patients (23.1% of referrals) did not attend the initial assessment appointment at the clinic despite a number of attempts to contact them and the clinic operating a flexible appointment system. Attendance for assessment did not vary significantly among different ethnic groups. Those patients who did not attend the MUS clinic also had significantly lower attendance rates at their GP practice (but no higher A&E attendance rates or specialist referral rates) in the previous year: mean = 7.8, s.d. = 4.8 *v*. patients who did attend the MUS clinic, mean = 13.65, s.d. = 10.50; *F* = 4.7, d.f. = 1, *P*<0.05.

There was no significant difference between patients attending/not attending in respect of age, gender, employment status, source of referral, presenting complaint, PHQ-15 scores, or with regard to a history of physical or mental disorder.

### Outcomes following MUS clinic assessment

Following assessment at the MUS clinic, 41 patients (36.2%) with a diagnosis of somatoform disorder were referred to BOPT; 34 patients (30.1%) were referred to secondary care mental health services and 13 patients (11.5%) were referred for other forms of psychological therapy, mainly cognitive-behavioural therapy (CBT). Only 11 patients (9.8%) were immediately referred back to GPs with specific advice to improve the clinical management. Fourteen patients (12%) were identified as not suitable for the MUS clinic, mostly due to possible medical causes not having been ruled out or because of physical symptoms identified as resulting from a psychotic presentation (i.e. cenesthesias or somatic hallucinations). Pharmacological interventions were initiated in the clinic as follows: starting antidepressants (*n* = 12); optimising the dose of existing antidepressant treatment (*n* = 13); and changing antidepressants or other psychotropic drugs (*n* = 10).

### BOPT group analysis

Out of the group of 41 patients identified with a primary diagnosis of somatoform disorder and referred for BOPT, only 12 (29.3%) participated in the assessment and treatment process (3 males (7.3%) and 9 females (22%)). The demographic and clinical characteristics of the group of patients undergoing BOPT did not differ significantly from those who did not participate. The mean age of participants was 43.5 years (s.d. = 6.2); distribution across ethnic groups was: Asian *n* = 8, White *n* = 2, African-Caribbean *n* = 2. Participants attended a mean of 11 out of 15 sessions offered (s.d = 5.2). Follow-up data were collected at 3 months after treatment for symptoms and functional scores. Data on service utilisation were collected for the year following the end of BOPT. The PHQ-15 score indicated significant improvement for somatic complaints following treatment but there was no significant difference in depression scores. Service utilisation in the year following BOPT (A&E attendance and referrals to specialist services) reduced significantly and GP attendance figures showed a considerable trend towards reduction in service use. Outcomes for the BOPT group are summarised in Table 2.

**Table 1:** Diagnostic group characteristics

	All patients	Somatoform disorders (F45)^a^	Depressive disorders (F32-34)^a^	Dissociative disorder (F44)^a^	Anxiety disorders (F40-43)^a^	Other disorders
Comorbidity	*n* = 30	Depressive disorder *n* = 12 (26%), anxiety disorder *n* = 5 (11%)	Anxiety disorder *n* = 5 (16%)	Depressive disorder *n* = 3 (75%)	Depressive disorder *n* = 3 (37%)	Depressive disorder *n* = 2 (18%)
*n* (%)	106	45 (42.5)	38 (35.8)	4 (3.8)	8 (7.5)	11 (10.4)
Age, years: mean (s.d.)		41 (9.1)^b^	48 (8.9)^b^^,^^c^	30.7 (7)^c^	42.3 (9.5)	39.5 (9)
						
Gender, *n* (%)	Male 34 (32), female 72 (68)	Male 13 (28.9), female 32 (71.1)	Male 11 (28.9), female 27 (71.1)	Male 1 (25), female 3 (75)	Male 1 (12.5), female 7 (87.5)	Male 8 (72.7)^d^, female 3 (27.3)
PHQ-15, mean (s.d.)	17.6 (6.7)	18.2 (7.1)	18.5 (6.4)	10.6 (4.9)	13.2 (2.4)	14.4 (8.4)
HRSD total, mean (s.d.)	18.4 (8.4)	16 (8.3)^e^	22 (6)^e^	15 (2)	7 (3.5)	19 (11)
SOMS-7 total, mean (s.d.)	52.7 (26.3)	49 (29)	53 (21)	38 (12)	36 (11)	66 (22)
GAF, mean (s.d.)	54.3 (13.4)	54.4 (15)	52.3 (12.5)	60 (15)	85 (10)	48 (18)

GAF, Global Assessment of Functioning; HRSD, Hamilton Rating Scale for Depression; PHQ-15, Patient Health Questionnaire; SOMS-7, Screening for Somatoform Symptoms; s.d. = standard deviation.

a. ICD-10 code primary diagnosis.

b. *Post hoc* test of mean difference between somatoform disorder and depression groups, *P*<0.01.

c. *Post hoc* test of mean difference between depression and dissociative disorder groups, *P*<0.01.

d. Comparison between other diagnostic group and rest of the cohort together, Pearson’s χ^2^ = 10, d.f. = 1, *P*<0.05.

e. *Post hoc* test of mean difference between somatoform disorder and depression groups, *P*<0.05.

## Discussion

### Factors influencing MUS liaison clinic service utilisation

The distribution of ethnic groups among patients who were referred to and attended the MUS clinic largely reflected the population statistics for the London borough of Newham.^17^ Asian patients presented more frequently with generalised pain than patients from other ethnic groups. Similar ethnic differences in expression of musculoskeletal pain have previously been reported in UK samples^22^ and this may be regarded as an unspecific but meaningful indicator for the prevalence of the somatoform syndrome in this group. This may also explain the discrepancy between the higher number of GP surgery visits and lower number of referrals to specialist services in the Asian group.

**Table 2:** Body-oriented psychological therapy group outcomes

Outcome measures	Before treatment Mean (s.d.)	3 months post-treatment Mean (s.d.)	Paired-sample *t*-test
HRSD total	19 (7.7)	18.2 (7.9)	*t* = 0.93, n.s.
PHQ-15 total	17.7 (3.3)	15.1 (4.9)	*t* = –2.2, *P*<0.05 95% CI 0.005 to 3.6
GP attendance	17.8 (10)	10.5 (7.8)	*t* = –2.0, n.s.
Specialist referrals	3.4 (2)	1.2 (1.5)	*t* = –5.7, *P*<0.01, 95% CI –3.1 to –1.4
A&E attendance	2.5 (1.9)	0.9 (0.7)	*t* = –2.8, *P*<0.05 95% CI –2.7 to –0.33

A&E, accident and emergency; GP, general practice; HRSD, Hamilton Rating Scale for Depression; PHQ-15, Patient Health Questionnaire; n.s., not significant.

It is noteworthy that the majority of patients assessed in the liaison clinic had a previously undetected mental disorder. More than a third of patients assessed for MUS had a significant depressive disorder (moderate to high HRSD scores) with somatic symptoms in the context of this primary diagnosis. This finding supports the inclusion of somatisation syndromes in depressive and anxiety disorders as referral criteria for MUS clinics, since patients with depression and anxiety may only present with somatic symptoms.^23,24^ It also emphasises the need for better recognition of depression in primary care as highlighted in National Institute for Health and Care Excellence guidelines.^25^

Pain localised to a particular area of the body was common in all diagnostic groups. The levels of somatic symptom severity and functional impairment were comparable across the cohort. This may be because of a bias towards referring mainly patients with high somatic symptoms from primary care, but it also emphasises the non-specific nature of somatic complaints as indicators of a variety of different mental disorders. While establishing a differential diagnosis for patients with MUS and in order to improve the recognition of undetected mental illness (depressive and anxiety disorders mainly), it seems necessary that specific teaching and assessment tools are more widely and routinely used among primary care practitioners.

### Service delivery for patients with MUS

Referral and access to the specialist MUS service across GP practices in the borough was clearly sporadic. This is despite a borough-wide campaign to raise awareness and facilitate the referral process. Only a small number of GPs made referrals to the service, possibly reflecting a personal special interest in the subject. There are, however, significant problems when discussing a biopsychosocial perspective with patients with MUS who predominantly seek an organic/medical explanation for their symptoms.

In terms of the patients presenting for the assessment process, nearly a quarter did not attend the MUS clinic; their somatic symptom severity was comparable to those who attended, suggesting a high level of unmet need in this area. Those patients who attended, who frequently attended GP surgery appointments, accepted the specialist MUS clinic setting within mental health services well, whereas the findings suggest that a significant proportion of patients with MUS may only be engaged successfully in a collaborative and creative management process offered at primary care level. The specialist clinic served as nodal point of expert advice for appropriate pharmacological and psychological intervention for patients with MUS. It also facilitated access to secondary care mental health services where appropriate.

### Association between depression and somatoform disorders and its implication

In the somatoform disorder group, the degree of depressive symptoms was the highest predictor for the overall level of patient function. This relationship held true even after controlling for comorbid clinical depressive episodes. This highlights the association of depressive symptoms with somatoform disorders, even at subsyndromal level. The small sample size of our cohort and methodological restrictions allow only for cautious interpretation, but the results confirm findings from previous studies regarding the close association between depression, anxiety and somatoform disorders and contribute to the renewed nosological discussion considering a significant overlap between them.^26-28^

### BOPT for MUS (somatoform disorder)

The results from the small sample of this pilot are in line with previous studies,^14,16,29-31^ indicating that BOPT is a promising intervention for patients with MUS, particularly those with undifferentiated somatoform pain disorder, who frequently attend primary care. There was an early reduction in the reporting of somatic symptom severity and also a significant reduction in service use for the year after therapy.

Other psychotherapeutic models successfully utilised for the treatment of MUS, such as brief psychodynamic interpersonal therapy and mentalisation-based CBT,^32,33^ equally refer to concepts of developmentally or learning-based dysregulations of bodily self-experience and relationships. The BOPT model, however, offers a fundamentally different approach, connecting cognitive and emotional levels with bodily states through enactment and expressive movement exercises. This is done without explicitly relating to potentially underlying psychological conflicts or identifying and modifying dysfunctional automatic thoughts. Hereby patients are enabled ‘to make the breakthrough to a new level of understanding, without the requirements of verbalization’.^34^ This treatment modality adds another option to the available spectrum of psychotherapeutic approaches.

Take-up of BOPT was relatively low, with only 12 out of 41 patients referred participating. This may be partially due to patients adopting somatically dominated explanatory models and their reluctance to engage in any form of psychological therapy, and/or because the therapy was provided within mental healthcare premises. Otherwise, it was encouraging to see that the body-oriented approach was mainly taken up by patients from an ethnic minority background.

### Strengths and limitations of the study

The study was conducted in a naturalistic setting as a service evaluation and the results are therefore mainly used to evaluate the feasibility and acceptability of such a service within the realms of primary-secondary care liaison services. The sample size and exploratory nature of the study only allow for a preliminary and cautious interpretation of the data. The study did not collect enough data on other potential confounding factors.

### Implications for future research or clinical practice

More specifically developed primary-secondary liaison services for patients with MUS problems are needed to improve the detection, recognition and adequate treatment of these patients and reduce the associated costs to healthcare systems. These services should include proactive identification (‘case finding’) elements and innovative models of engagement with patients within primary care settings (e.g. joint consultation model).

More research is needed to identify suitable service pathways and placement of service delivery, in a way that would provide better access to adequate care for all patients with MUS.^35^ The recently published Dutch multidisciplinary guideline for MUS similarly emphasises the importance of a ‘disease-management based on risk profiles, providing stepped care and case management by the GP, supported by psychiatric consultation’.^36^ For the UK, the development of innovative new treatments has been encouraged in the context of the expanded Improving Access to Psychological Therapies (IAPT) pathfinder programme for people with long-term conditions and/or MUS.

The significant reduction in symptom levels and service utilisation in the small BOPT group described here seems promising. This impact should be evaluated in an adequately powered and larger controlled study aiming specifically to explore the benefits of BOPT for patients who otherwise do not engage in or do not respond to talking therapies. The new intervention could potentially translate into significant cost savings to the health service if this approach can demonstrate cost-effectiveness in larger trials.
